# Impact of an Individualized Physical Activity Intervention on Improving Mental Health Outcomes in Family Caregivers of Persons with Dementia: A Randomized Controlled Trial

**DOI:** 10.3934/medsci.2016.1.15

**Published:** 2015-12-17

**Authors:** Carol J. Farran, Olimpia Paun, Fawn Cothran, Caryn D. Etkin, Kumar B. Rajan, Amy Eisenstein, Maryam Navaie

**Affiliations:** 1 Rush University Medical Center, College of Nursing, Chicago, 1L, USA; 2 American Joint Replacement Registry, Rosemont, IL, USA; 3 Rush University Medical Center, Department of Internal Medicine, Chicago, IL USA; 4 CJE Senior Life, Chicago, IL, USA; 5 Meridian Health, Office of Research Services, Neptune, NJ, USA

**Keywords:** Alzheimer’s disease, emotional well-being, health promotion interventions, family caregivers

## Abstract

**Purpose:**

This study examined secondary benefits of an individualized physical activity intervention on improving dementia family caregivers’ subjective burden, depressive symptoms and positive affect.

**Design and Methods:**

A community-based randomized controlled trial (RCT) was implemented with family caregivers of persons with dementia (N = 211) who received the Enhanced Physical Activity Intervention (EPAI: treatment intervention, n = 106) or the Caregiver Skill Building Intervention (CSBI: control intervention, n = 105). Interventions were delivered over 12 months, including a baseline home visit and regularly spaced telephone calls. Data were collected in person at baseline, 6 and 12-months; and telephonically at 3 and 9-months. The EPAI integrated physical activity and caregiving content while the CSBI focused only on caregiving content. Descriptive, bivariate and intention-to-treat analyses using generalized estimating equations (GEE) were performed to examine secondary benefits of the EPAI on family caregiver burden, depressive symptoms and positive affect.

**Results:**

Compared to caregivers in the CSBI group, caregivers in the EPAI significantly increased their overall and total moderate physical activity and showed a positive interaction between the intervention and time for positive affect at both six (*p* = 0.01) and 12-months (*p* = 0.03). The EPAI was significantly associated with improving burden at 3 months (*p* = 0.03) but had no significant effect on depressive symptoms.

**Implications:**

Caregiver involvement in an individualized physical activity intervention was associated with increased overall and total moderate physical activity and improved positive affect from baseline to 12 months. Improved positive affect may help caregivers to feel better about themselves and their situation, and better enable them to continue providing care for their family member for a longer time at lower risk to their own mental health.

## 1. Introduction

Dementia is a neurocognitive disorder with Alzheimer’s disease being the most common cause. Dementia affects the part of the brain that interferes with a person’s independence in daily activities. It is estimated that 5.3 million Americans of all ages have Alzheimer’s disease (AD) or a related dementia, and that in 2015, caregivers of these persons provided an estimated 17.9 billion hours of uncompensated care [1]. Economically, the value of care provided by family caregivers has been estimated to be $217.7 billion annually [1].

Over the past 30 years, more than 200 psychoeducational randomized controlled trials have been conducted with dementia family caregivers and generally, these interventions have been effective [2]. Common intervention outcomes have included family caregivers’ appraisal of how much burden they experienced, how well they were coping and whether they felt they could manage their relative’s behavioral symptoms [3]. On average, these studies showed statistically significant effect sizes for improvement of depression, caregiver burden and subjective well-being that ranged from 0.14 to 0.41 standard deviation units [4]. However, Schulz et al. [5] have suggested that greater emphasis should be placed on outcomes viewed in a public health context that have potentially far-reaching consequences for promoting caregiver health and wellness. Increasing caregiver physical activity is one such possible intervention for improving caregiver health.

### 1.1. Caregiver burden

Caregiver burden is conceptualized as a multidimensional response to the negative appraisal and perceived stress associated with caring for the care recipient [6]. Up to 40% of caregivers report experiencing burden [7]. Objective burden assesses both negative and upsetting experiences while subjective burden focuses on perceptions of distress over particular experiences. Burden may affect how caregivers interact with others, as well as other roles they assume outside their home [8]. Caregiver burden has been associated with poorer mental health and worse physical health outcomes; and where caregiver’s mental health declines, it may result in their relative’s self-neglect and diminished quality of care [9]. Risk factors for caregiver burden include being female, older, living with the care recipient, providing more hours of care, having diminished caregiver physical health, or feelings of ‘not having a choice’ in becoming a caregiver [10]. Positive social interactions and affection have been reported as playing a role in reducing caregiver burden [11]. Limitations of burden research include inconsistent measurements, as well as predominantly using cross-sectional designs [12,13].

### 1.2. Caregiver depressive symptoms

Caregiver depressive symptoms are well documented effects of dementia caregiving [14,15]. A caregiver meta-analysis determined that although 51% of studies reported reduction of caregiver depressive symptoms, there were no statistically significant effects on depressive symptoms in studies that exclusively enrolled dementia caregivers [16]. Although there is consensus that dementia caregivers experience depressive symptoms, interventions targeting mental health outcomes have had mixed results, with some studies suggesting modest to no significant results, while others have reported both 8- and 24-month positive outcomes [17]. In general, evidence supports the use of highly individualized interventions that specifically target dementia caregivers’ depressive symptoms [18].

### 1.3. Positive aspects of caregiving

Considerably fewer studies have focused on positive family caregiver outcomes [19–21]. Positive affect, viewed as the opposite of negative affect, reflects a spectrum of pleasant states, attitudes and well-being [22,23]. Adapting negative thoughts and feelings may help caregivers improve how they feel about their situation [24,25]. Positive affect has been associated with fewer sleep problems in older caregivers but not non-caregivers [22]; with caregiver positive perceptions following care recipient attendance at adult day care [26]; and with a caregiver intervention that delayed nursing home placement [20]. Multicultural differences in positive affect have been noted, with African Americans reporting that caregiving gave them “a more positive attitude toward life” and enabled them to “appreciate life more” than either Whites or Hispanics [21]. However, research on caregiver positive aspects has been limited by inconsistent conceptualization and measurement [25].

### 1.4. Physical activity studies

Numerous population-based prospective cohort studies provide substantial evidence that active people on average had more than 30% lower odds of feeling distress, 45% lower odds of depressive symptoms, and 30% higher odds of experiencing enhanced well-being than inactive persons. Moderate and high levels of physical activity reduce the odds of developing depressive symptoms compared to low levels of physical activity exposure. Both moderate and high levels of physical activity are more protective of greater health than very low levels of physical activity or inactivity. Optimal type or amount of exercise for reducing depressive symptoms is not clearly identified, although it appears that an increase in physical fitness is not required. Overall, higher amounts of physical activity have been associated with a broader spectrum of health benefits [27]. Therefore in the context of stressful situations, such as caregiving, maintaining physical activity may be beneficial.

### 1.5. Caregiver physical activity interventions

Few trials have tested interventions designed to increase caregiver physical activity. This is a concern, given that activity guidelines are met by fewer than 50% of adults age > 65 years and fewer than 36% of women—despite known psychological and physical benefits [27]. Among family caregivers, as few as 20% report engaging in vigorous physical activity for at least 20 minutes three times/week. Although many psychoeducational caregiver interventions have been conducted [28], far fewer caregiver physical activity interventions have been tested. The few that have been reported note that physical activity interventions are feasible, they improved caregiver self-efficacy and physical activity, decreased psychological distress [29]; and improved caregiver energy expenditure, stress-induced blood pressure reactivity and sleep [30,31]. A recent systematic review of these three studies, using pooled data, noted that physical activity was favored in reducing subjective caregiver burden, using two different burden measures; but there was no observable effect of physical activity on objective caregiver burden, depressive symptoms or perceived stressors [32].

### 1.6. Physical activity studies with other older adults

Other physical activity studies conducted with older adults, similar in age to dementia caregivers, noted that at 12 months higher levels of self-efficacy and positive affect were associated with higher quality of life. At 48 months, changes in physical activity were related to increased self-esteem and positive affect, but only positive affect directly influenced improvements in quality of life. These findings suggested that physical activity effects on quality of life were, in part, mediated by intermediate psychological outcomes such as positive affect, and may potentially have a longitudinal effect on family caregiver quality of life [33,34].

## 2. Study Overview

An initial physical activity pilot study demonstrated feasibility and preliminary intervention effectiveness in increasing caregiver lifestyle physical activity [35]. The study reported herein, was part of this larger RCT that used a pre-test-multiple post-test design to test the effectiveness of the Enhanced Physical Activity Intervention (EPAI: treatment condition) to increase total and total moderate physical activity in comparison to the Caregiver Skill Building Intervention (CSBI: control condition) [Farran, Etkin & Eisenstein, unpublished data]. The study followed the National Institutes of Health Randomized Clinical Trials Involving Behavioral Interventions Guidelines [36]. The purpose of this 12-month secondary RCT analysis was to determine if the EPAI was more effective than the CSBI in improving three caregiver mental health outcomes: (1) Perceived burden, a stress appraisal variable, (2) Depressive symptoms, a mental health response to stress, and (3) Positive affect, a process or mechanism by which caregivers change their perception of caregiving.

## 3. Materials and Methods

### 3.1. Study design

This secondary analysis used data from an RCT that tested effectiveness of the Enhancing Physical Activity Intervention (EPAI: treatment group) in comparison to the Caregiver Skill Building (CSBI: control group). A total of 325 family caregivers of persons with dementia were assessed for eligibility, with 211 caregivers randomly assigned to one of two study arms (EPAI, n = 106; CSBI, n = 105).

### 3.2. Power analysis

A sample size of 190 was determined from our a priori hypothesis which consisted of a two-way comparison between the two groups for increasing weekly minutes of total physical activity. For a Type I error rate of 0.05 and a one-sided test, it was estimated that we would have 80% power to detect a standardized difference (effect size) of 0.395 in increasing weekly minutes of physical activity between the EPAI and CSBI. Adherence to the study varied by intervention group with 63% of EPAI in comparison to 84% of CSBI caregivers completing the trial, for an average of 74%. As the study progressed, we observed this differential EPAI attrition so we recruited 21 additional caregivers (N = 211) [Farran, Etkin & Eisenstein, unpublished data].

### 3.3. Selection, baseline assessment and randomization of caregiver participants

Participants who met the following inclusion criteria were recruited by the study coordinator: (1) family caregiver of a person diagnosed with probable or possible AD, or a related dementia who resided at home; (2) ≥ 30 years of age and English speaking; (3) reported some to moderate strain in providing care for ≥ 1 personal/instrumental activities of daily living [37]; (4) provided ≥ 10 hours of care per week; (5) not planning to relocate or place care recipient in a nursing home within 6 months; (6) had regular telephone access; (7) engaged in ≤ 60 minutes of regular physical activity per week for ≥ 6 months; (8) not involved in another caregiver intervention; and (9) had no medical conditions contraindicating moderate physical activity participation.

### 3.4. Randomization

The study coordinator confirmed caregiver (CG) eligibility using data management reports. If the inclusion criteria were met and the CG expressed interest in study participation, a baseline home interview was scheduled by one of two research assistants (RA). During the baseline visit, the RA obtained informed consent and administered the CG baseline assessment. Procedures were fully specified and data were collected via computer-based direct entry, limiting missing data. Once baseline data were complete, the study coordinator randomly assigned subjects on an ongoing basis, to one of two study arms: the EPAI (treatment condition) or the CSBI (control condition), using a computerized-generated list of numbers prepared by the statistician (1’s and 2’s) in blocks of 15 subjects every 3 months. This list was balanced with approximately 7–8 persons in each group, to assure equal assignment to groups for practical and administrative reasons, and avoided temporal influence on the intervention. The treatment allocation was concealed from caregivers and their family members.

### 3.5. Overview of interventions

Both the EPAI and CSBI were guided by Bandura’s social cognitive theoretical model [24] and used an identical structure and process that totaled 20 planned contacts. Weekly intervention contacts were provided from baseline to 3 months, bimonthly contacts from 4 to 6 months, and monthly contacts from 6 to 12 months. The baseline interview was an in-home visit, as were the 6- and 12-month visits. The week 2- through week 12- intervention visits and 3- and 9-month assessment visits were all completed by telephone. Each intervention had its own treatment manual and was implemented by an individually assigned PhD-prepared interventionist.

The EPAI goal was to increase lifestyle physical activity and address standard care-related issues that may function as barriers to physical activity participation. EPAI content areas included: (1) Getting started, (2) What physical activity can do for me, (3) My current routine, (4) Developing a plan for increasing physical activity, (5) How to keep going, (6) Building a strong support system, (7) Setting goals for months 5–12, and (8) Individual concerns. EPAI content addressed caregiving-related barriers that could interfere with physical activity participation, such as care recipients’ personal activities of daily living, behavioral symptoms of dementia and caregiver self-care. The EPAI physical activity component was tailored to individual family caregiver’s needs based upon their interests and abilities concerning the type, frequency, intensity and duration of physical activity [30]. Caregivers used a pedometer to assess their level of physical activity and selected a combination of physical activities that were appropriate to their abilities; and set goals for moderate physical activities of > 150 minutes/week. Once they met moderate physical activity goals, they set new goals that included stretching, balance, and/or strength building.

The CSBI goal was to help caregivers understand how to meet basic needs related to dementia care. This intervention was designed to minimize treatment exposure to physical activity and was tailored after usual-care interventions [38]. Specific content focused on: (1) Getting started, (2) Understanding dementia and safety issues, (3) Providing family member personal care, (4) Handling difficult behaviors, (5) Managing caregiver stress, (6) Finding and using services, and (7,8) Facing individual concerns.

### 3.6. Ethical approval and consent

Rush College of Nursing served as the primary study site and was involved in overall study management, participant recruitment, obtaining caregiver study consent, intervention implementation, and data analysis and dissemination. This RCT was approved by the Rush University Medical Center Internal Review Board and the study was pre-registered with ClinicalTrials.gov: NCT00721383.

### 3.7. Data collection procedures and measures

Two well-trained research assistants who were blind to treatment assignment, collected data at baseline, 3, 6, 9, and 12 months. In-home assessments occurred at baseline, 6 and 12 months; telephone assessments occurred at 3 and 9 months. Data were collected using BLAISE, a Pascal-based, commercially available software package to create an entry platform for all interview procedures. Programming required the design of on-screen displays, specification of branching patterns, range and contingency checks, and setting up linkages to schedule and monitor study management databases. This program had the advantages of: rapid data acquisition, reduced data processing costs, reduced missing or inaccurately coded data due to online logic checks, electronic capture of interviewer question-related comments, and increased efficiency of scheduling and monitoring progress necessary for frequent sequential measurement. Both caregivers and care recipients were blind to treatment assignment.

Measures included: (1) caregiver and care recipient sociodemographic characteristics specific to age, gender, marital status, employment, level of education, relationship to care recipient, living arrangements, racial characteristics [39]; and the care recipient’s Mini Mental State Examination score [40], and (2) the Community Health Activities Model Program for Seniors (CHAMPS) measure which includes caregiver self-reported total and total moderate physical activity (defined as > 150 minutes/week), in a typical week in the past month [41]. (3) Intervention Implementation was assessed by the total number of sessions in which caregivers participated. Each intervention was monitored by two different PhD-prepared clinician/scientists who reviewed taped intervention sessions and regularly met with their specified interventionist for support and to address challenges to protocol implementation.

Caregiver mental health was assessed using three standardized instruments: (1) **Perceived burden** was measured by the 11-item Subjective Caregiving Burden Scale [23]. Seven items focused on caregiver feelings as a result of providing care: not having enough time for self, personal health has suffered because of caring for my relative, social life has suffered, feel isolated and alone, feel unable to care for family member much longer, have lost control since CR’s (care recipient’s) illness, and feel very tired because of caregiving. Item responses ranged from 0 = *never* to 4 = *nearly always.* Four remaining items focused on caregiver feelings about providing care (i.e., 1 can fit in most things 1 need to do in spite of caring for CR, it is hard to plan ahead due to CR’s unpredictable needs, CR needs determine how my days are spent, caring for CR gives me a trapped feeling). Responses to these items ranged from 0 = *disagree* a lot to 4 = *agree a lot* (range for total measure = 0–44). Cronbach’s coefficient alpha as an assessment of internal consistency of the measure with this sample was 0.87; (2) **Depressive symptoms** were measured with the 10-item Center for Epidemiologic Studies Depression Scale (CES-D) [42]. Items focused on feelings (i.e., depressed, everything takes an effort, restless sleep, happy, lonely, unfriendly, enjoy life, sad, people dislike me, unable to ‘get going’). Responses included: 1 = *yes* and 0 = *no* (range = 0–10). Cronbach’s coefficient alpha for internal consistency/reliability with this sample was 0.92; and (3) **Positive affect** was measured using the positive affect subscale of the Positive and Negative Affect Scales (PANAS), which include 10 items that focused on how interested, excited, strong, enthusiastic, proud, alert, inspired, determined, attentive and active that caregivers were [43]. Responses ranged from 1 *- very slightly or not at all* to 5 = *extremely* (range = 10–50). Cronbach’s coefficient alpha for internal consistency/reliability with this sample was 0.91.

## 4. Data Analysis

Descriptive and bivariate analyses were performed for comparative purposes using chi-square and Student’s t-test, with *p* < 0.05 denoting statistical significance. Intention-to-treat analyses were used to examine study results. To permit full use of longitudinal data, generalized linear mixed models were employed using the generalized estimating equation (GEE) approach to evaluate 12-month EPAI effects on improving caregiver mental health outcomes (i.e., Burden, Depressive symptoms and Positive affect). Analyses were conducted using SAS 9.3 [44]. To determine if there were differences between study completers and study drop-outs, comparative analyses using Student’s *t*-tests and chi-square analyses were examined. If there were differences between these two groups, these variables were controlled for in subsequent analyses.

## 5. Results

The average age of study participants was M = 62 years old (SD = 13). The majority was women (82%), married (63%), and not employed (63%). About a quarter included college graduates or had masters/doctoral degrees (26%). Family caregivers were almost equally divided between being adult children (50%) or spousal caregivers (44%), and the majority lived with their impaired family member (89%). The majority were non-Hispanic white (66%), African American (27%), or of Hispanic or other origin (8%). Care recipients were M = 80 years old (SD = 19); 64% were women and they were moderately impaired with a Mini Mental State Exam (MMSE) M = 16 (SD = 8) [40], No baseline sociodemographic differences were found between the EPAI and CSBI, suggesting that randomization was effective (Farran, Etkin & Eisenstein, unpublished data).

Overall and total moderate physical activities were examined, using Student’s t-tests and GEE from baseline and 12-months (Tables 1 & 2). CSBI caregivers reported significantly higher levels of total physical activity at baseline than the EPAI (*p* = 0.02) (Table 1). However, at 12 months, EPAI caregivers reported significantly higher number of total physical activity minutes (*p* = 0.03), having increased their overall physical activity by +141 minutes/week while CSBI participants reported decreasing their overall physical activity by −124 minutes. EPAI caregivers also reported significantly more total moderate physical activity minutes from baseline to 12 months—increasing this level of physical activity minutes/week by +41 minutes—while CSBI participants decreased their total moderate physical activity by −70 minutes (*p* = 0.03).

### 5.1. Intervention adherence

Caregivers could participate in up to 20 intervention sessions. CSBI caregivers participated in an average of 18 sessions (90% adherence to the intervention), while EPAI caregivers participated in an average of 14 sessions (*p* = 0.01) (70% adherence to the intervention), for an overall average intervention adherence of 80%. There were no significant differences by intervention group for the total time in which caregivers spent in each intervention (*p* = 0.07) (Table 1).

### 5.2. Mental health outcomes

*Results from Student’s t-tests* examined differences between the two interventions for 12-month mental health outcomes (i.e., Burden, Depressive symptoms and Positive affect). No significant mean differences between the EPAI and CSBI for any of the mental health variables at either baseline or 12 months were noted (Table 1).

To address mental health change over time, GEE models examined three effects for each mental health variable: study time (3–12 months), main effect of group (EPAI), and the EPAI-by-study time interaction (Table 2). For Perceived burden, there was a significant interaction between the EPAI-by-study time at month 3 (*p =* 0.03); but for Depressive symptoms there were no significant interactions between the EPAI by any study month. For Positive affect, there were significant interactions between the EPAI-by study-month at both 6 and 12 months. These estimates for Positive affect (i.e., 2.76 and 2.30) suggested that EPAI Positive affect increased by ≥ 2 points at both 6 and 12 months.

**Figure 1:**
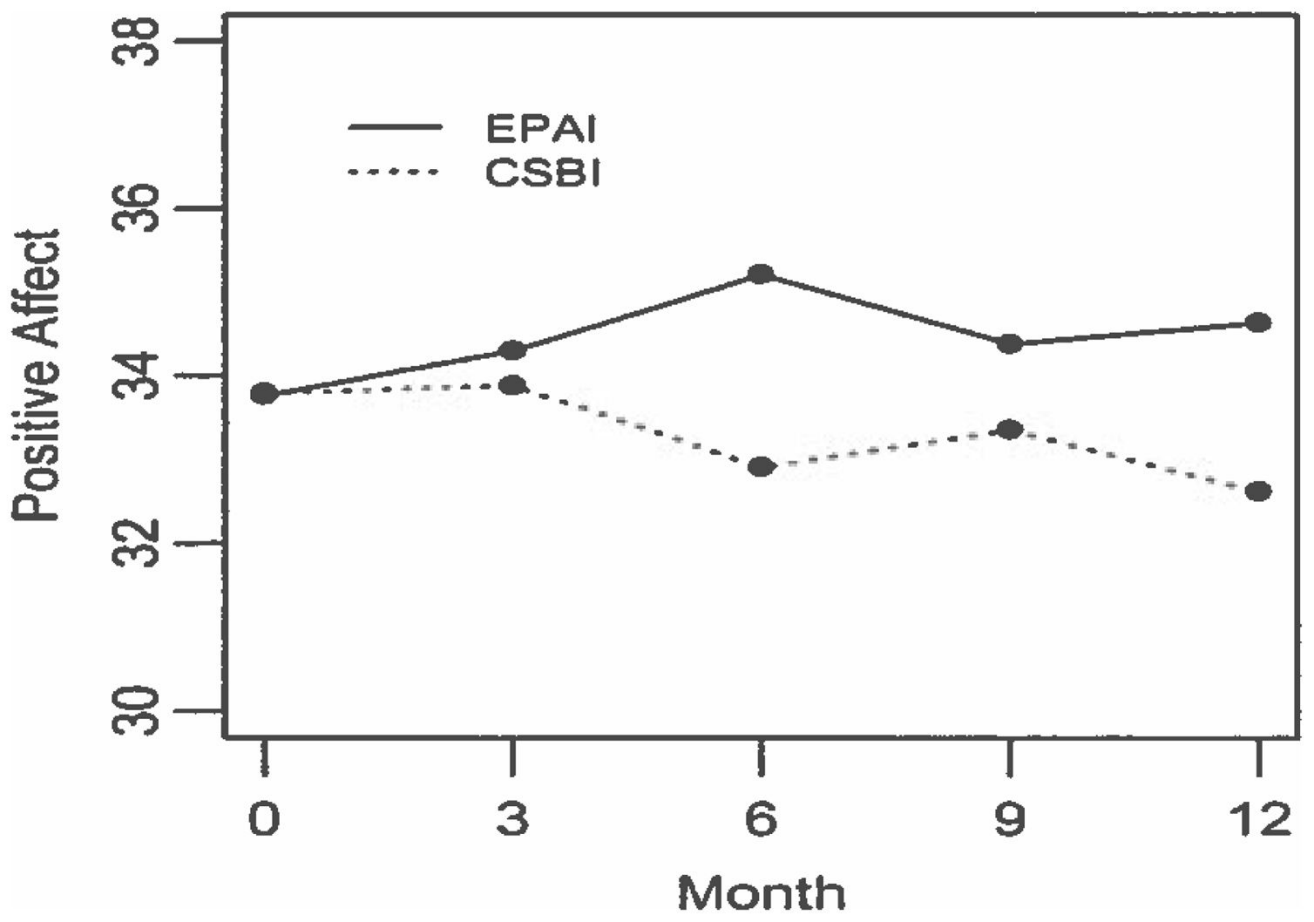
Changes in Positive Affect from Baseline to 12 months

Of the three mental health outcomes, only Positive affect showed a significant EPAI-by study time interaction at both 6 and 12 months when data were collected in-person (*p* = 0.01 and 0.03, respectively), as opposed to months 3 and 9 when they were collected by telephone (*p =* 0.64 and 0.54) (Figure 1).

## 6. Discussion

The purpose of this secondary analysis of RCT data was to determine if the EPAI was effective in improving caregiver mental health, using three common caregiver measures: Perceived burden, a stress appraisal measure; Depressive symptoms, a mental health response to stress; and Positive affect, a process or mechanism by which caregivers adapt their perception of caregiving. For these mental health outcomes, there were no significant mean differences between the EPAI and CSBI at baseline or 12 months. However, GEE multivariate models, which provided the advantage of using more study observations over time, showed that for Positive affect, there were significant interactions between the EPAI and increased Positive affect at both 6 and 12 months. For Perceived burden, there was only a significant interaction between the EPAI and time at month 3; while for Depressive symptoms, there were no significant interactions between the EPAI and time at any month. A prior meta-analysis of four known caregiver physical activity interventions determined that these studies were effective in reducing caregiver subjective burden using two different burden measures. However, these studies did not show that physical activity had an effect on caregiver perceived stress, depressive symptoms or anxiety. Limitations of these studies included the quality of the evidence and the small number of RCTs [32]

Positive affect has been described as a process or mechanism by which persons change their perception. We hypothesized that as a result of the EPAI, caregivers changed their behavior and perception about several intervention-related variables: by increasing their overall and total moderate physical activity and significantly increasing their positive affect. We surmised that positive affect changes were due to the EPAI’s social cognitive theoretical approaches that guided this intervention [24]. Throughout this intervention, caregivers learned more about (1) self-regulation—setting personal physical activity goals, structuring their outcome expectations and using self-rewards and other reinforcement strategies as they met their physical activity goals; (2) behavioral rehearsal—self-monitoring their physical activity and identifying barriers such as caring for their relative that might interfere in meeting physical activity goals; (3) reciprocal determination—they were guided to being more aware of environmental influences that helped or hindered them in meeting physical activity goals, and relied upon the interventionist and possibly other social support networks and affiliations, who encouraged these healthy behaviors; (4) self-reflection which may have helped them to identify personal thoughts, feelings and beliefs about physical activity and family caregiving; and (5) they were encouraged to be open to vicarious learning where they learned new things about themselves and identified positive changes that may have occurred as a result of increasing their physical activity and increased awareness about their caregiving responsibilities. Physical activity was initially evidenced by significant increases in both overall and total moderate physical activity; and these data supported that caregiver increased physical activity and improved Positive affect occurred only in the EPAI [27]. These findings were further supported by physical activity research conducted with older adults by McAuley and colleagues [33,34] who found that, at 1-year post-intervention, physical activity was related to positive variables such as self-efficacy, physical self-esteem and Positive affect, and in turn, greater levels of self-efficacy and Positive affect were associated with higher levels of quality of life. Other caregiver studies have addressed the importance of including Positive affect as a study variable for its association with caregiver fewer sleep problems [30]; and care recipient attendance in adult day care and delayed nursing home placement [20,26], while other physical activity studies reported well-being as a possible secondary outcome [33,34].

The interaction between Positive affect and time at both 6 and 12 months occurred when the measure was administered in-person, but not at months 3 and 9 when the measure was administered by telephone. It is possible that the PAN AS was more sensitive to eliciting positive feelings when administered in-person as opposed to by telephone, which may have seemed more impersonal to family caregivers [45,46]. This finding is similar to that in epidemiological research where face-to-face interactions were found to be more effective than less personal approaches such as group or telephone contacts [47]. In-person data collection may also have increased the potential for response bias [46,47].

Why did subjective Perceived burden and Depressive symptoms not significantly decrease over 12 months? These findings were inconsistent with a number of earlier physical activity studies that demonstrated a reduction in subjective burden [29]. Caregiver burden is closely associated with the amount of care provided by the caregiver for personal activities of daily living and behavioral symptoms of dementia [23]. These care-related tasks are not likely to change even if caregivers engaged in more physical activity because burden is more closely related to these care-related issues while physical activity is more likely associated with *behavioral changes,* which caregivers need to make when increasing their physical activity [24]. Further, it is possible that other caregiver Perceived burden could by its nature, be unaffected by the intervention. Such Perceived burden could be indirectly related to care provision and other variables (i.e., sociodemographic variables such as caregiver age, culture and relationship to the care recipient; or lack of resources such as inadequate finances, social support, unwanted caregiver role, or lack of confidence in caregiver skill). These variables may also have contributed to these findings as they would not be likely to change in the presence of participation in a physical activity intervention [10,11],

The lack of a significant effect on caregivers’ Depressive symptoms over 12 months can be explained by other existing caregiver research. A systematic review of 44 dementia caregiver intervention studies [18] determined that most types of psychosocial interventions were ineffective in significantly decreasing caregivers’ Depressive symptoms. The only studies that reported significant effects on dementia caregivers’ Depressive symptoms used a group-based psychosocial intervention designed with a specific focus on depression [18]. Neither the EPAI nor CSBI had depression/depressive symptoms as their main focus and both were implemented using an individual, rather than a group intervention approach. Study findings suggested that physical activity alone did not directly contribute to a significant decrease in Depressive symptoms in these dementia caregivers.

To reiterate, study results did not support that increasing physical activity reduced Perceived burden or Depressive symptoms, as reflected in past studies [29,45,48]. A physical activity intervention may not have a direct effect on caregiving burden as a stress appraisal variable, even though caregiving issues that might have been barriers to increasing physical activity were addressed. Similarly, the lack of significant intervention differences in Depressive symptoms may have been attributable to several factors: (1) there was content overlap between the EPAI and CSBI as both interventions addressed caregiving content and interventions likely were not significantly different concerning this content, (2) caregivers’ low baseline levels of depressive symptoms may also have contributed to the absence of a decrease in these symptoms over the 12-month follow-up period, and (3) the physical activity intensity may have been insufficient to demonstrate improvement in burden and depressive symptoms.

Study findings must also be viewed in light of other limitations. First, we do not know the impact on study outcomes of differential attrition in the EPAI compared to CSBI [Farran, Etkin & Eisenstein, unpublished data]. Second, level of physical activity was self-reported upon screening and again upon baseline, 6 and 12 months. Although caregivers reported participating ≤ 60 minutes of regular physical activity for ≥ 6 months upon screening, they reported considerably higher levels of physical activity when assessed using the CHAMPS [41]. This discrepancy in reporting physical activity may be attributed to several possible reasons. Upon recruitment, caregivers may have under-reported their physical activity because they *wanted* to participate in the study. Another possibility is that the CHAMPS (assessed at baseline, 6 and 12 months) may have resulted in higher ratings because it includes a broader variety of overall and moderate physical activities that caregivers may not initially have considered. Also, caregiver regular use and reporting of pedometer steps during EPAI sessions supported our confidence in the reliability of their CHAMPS physical activity assessments. In addition, results did not articulate the required physical activity therapeutic dose needed to improve caregiver mental health. Lastly, results may not be generalized to other caregiver populations. Despite these limitations, this study expands the existing knowledge base concerning a caregiver physical activity intervention and is a feasible intervention targeted specifically to stressfull dementia caregivers that may have an impact on their positive affect and potentially, caregiver quality of life [33,34].

## 7. Conclusions

This study adds to the limited body of research on physical activity interventions with family caregivers on several levels [29–32,35,49]. First, results support earlier studies that indicate that stressed family caregivers were able to increase their physical activity [29,31,49] as well as improve their positive emotional outlook. Second, this study demonstrated the feasibility of implementing an individualized, flexible physical activity intervention that combined both physical activity and caregiving-related issues, and following caregivers for 12-months. And third, the study theoretically supported that future intervention studies should give more attention to helping caregivers use a social cognitive/behavioral approach, with a greater focus on positive appraisals and re-appraisals [33,34]. Moreover, increasing positive affect may be the single most important finding in this study. Most interventions focus on decreasing potentially “negative” responses to caregiving, such as burden and depressive symptoms [7], while fewer focus on how caregivers can be assisted to feel more positive about their situation. Helping caregivers to do ‘something positive’ for themselves, such as physical activity, may help them feel better not only about themselves but also their caregiving situation on a day-to-day basis, and ultimately, may help caregivers to maintain their caregiving roles for longer periods at lower risk to their own mental health and quality of life [33,34].

Recommendations for future caregiver physical activity intervention studies include an emphasis on: (1) building the intervention upon a strong social cognitive theoretical basis that helps caregivers to learn how to better change their behaviors concerning physical activity and family caregiving [24,43]; (2) including specific intervention content and approaches that focus on helping caregivers to learn how to reframe and positively interpret what is happening in their lives and build upon a health-promoting lifestyle that may further help them to meet their caregiving demands [3], while at the same time caring for themselves; (3) evaluating a subgroup of caregivers who scored in the depression range; (4) considering future analyses which examine extent to which level of total and/or moderate physical activity were associated with caregiver mental health outcomes; and (5) adding health economics data to examine the long-term effects of a physical activity intervention on caregiver health-related quality of life and quality-adjusted life-years (QALYs) [50,51].

## Figures and Tables

**Table 1:** Baseline and 12-Month differences in physical activity, intervention implementation and mental health outcomes by intervention group.

Variables	EPAI n=106	CSBI n=105	*P*
***CHAMPS Self-Report Physical Activity***			
Total Physical Activity minutes/week			
Baseline	670±512	762 ±651	0.02
12 months (EPAI n = 53, CSBI n = 73)	811 ±534	638 ±395	0.02
Moderate Physical Activity minutes/week			
Baseline	207 ±377	193 ±279	0.001
12 months (EPAI n = 53, CSBI n = 73)	248 ±275	123 ±207	0.03
***Intervention Implementation*** (Total number of sessions = 20)			
Total sessions attended (M ± SD)	14±6	18±5	0.01
Total intervention time (minutes) (M ± SD)	354±166	383±123	0.07
***Mental Health Outcomes***			
Perceived burden			
Baseline	20.6 ±8.8	20.9 ±9.2	0.82
12 months (EPAI n = 53, CSBI n = 72)	19.8 ±8.8	19.4 ±9.4	0.84
Depressive Symptoms			
Baseline	3.4 ± 2.6	3.2 ±2.4	0.47
12 months (EPAI n = 53, CSBI n = 72)	2.6 ±2.3	2.9 ±2.4	0.60
Positive Affect			
Baseline	33.8 ±8.1	34 ±8.1	0.99
12 months (EPAI n = 53, CSBI n = 72)	34.6 ± 7.5	32.6 ±8.1	0.16

Note.

a Enhancing Physical Activity Intervention;

b Caregiver Skill Building Intervention.

**Table 2:** Mental health change over time for caregiver burden, depressive symptoms and positive affect by time, main effect and group-by-time interaction: baseline to 12 months.

Parameter	Estimate	Stnd Error	*P*	Estimate	Stnd Error	*p*	Estimate	Stnd Error	*P*

	*Perceived Burden*			*Depressive Symptoms*			*Positive Affect*		
# Observations		773			769			774	
Baseline	20.87	0.89	< 0.0001	1.14	0.07	< 0.0001	33.80	0.78	<0.0001
month3	−0.09	0.56	0.87	−0.09	0.08	0.24	−0.06	0.65	0.93
month6	−0.48	0.60	0.43	−0.00	0.07	0.96	−1.50	0.56	0.01
month9	0.10	0.65	0.88	−0.19	0.09	0.04	−0.44	0.74	0.55
month12	−0.53	0.62	0.39	−0.02	0.08	0.81	−1.50	0.77	0.05
epai	−0.38	1.24	0.76	0.08	0.11	0.44	0.03	1.11	0.98
epai_month3	−1.72	0.81	0.03	−0.08	0.11	0.46	0.45	0.96	0.64
epai_month6	−0.85	0.93	0.36	−0.16	0.10	0.10	2.76	0.98	0.01
epai_month9	−0.51	0.98	0.60	−0.07	0.12	053	0.68	1.12	0.54
epai month 12	−0.09	0.98	0.92	−0.18	0.11	0.11	2.30	1.03	0.03

## Abbreviations

AD: Alzheimer’s disease and other dementias
CES-D: Center for Epidemiologic Studies-Depression Scale
CSBI: Caregiver skill building intervention
CR: Care recipient
EPAI: Enhancing physical activity intervention
GEE: Generalized estimating equations
IADL: Instrumental activities of daily living
MMSE: Mini Mental State Exam
PANAS: Positive and Negative Affect Scales
RCT: Randomized controlled trial
SAS: Statistical Analysis System
TRAC Study: Telephone Resources and Assistance for Caregivers Study
